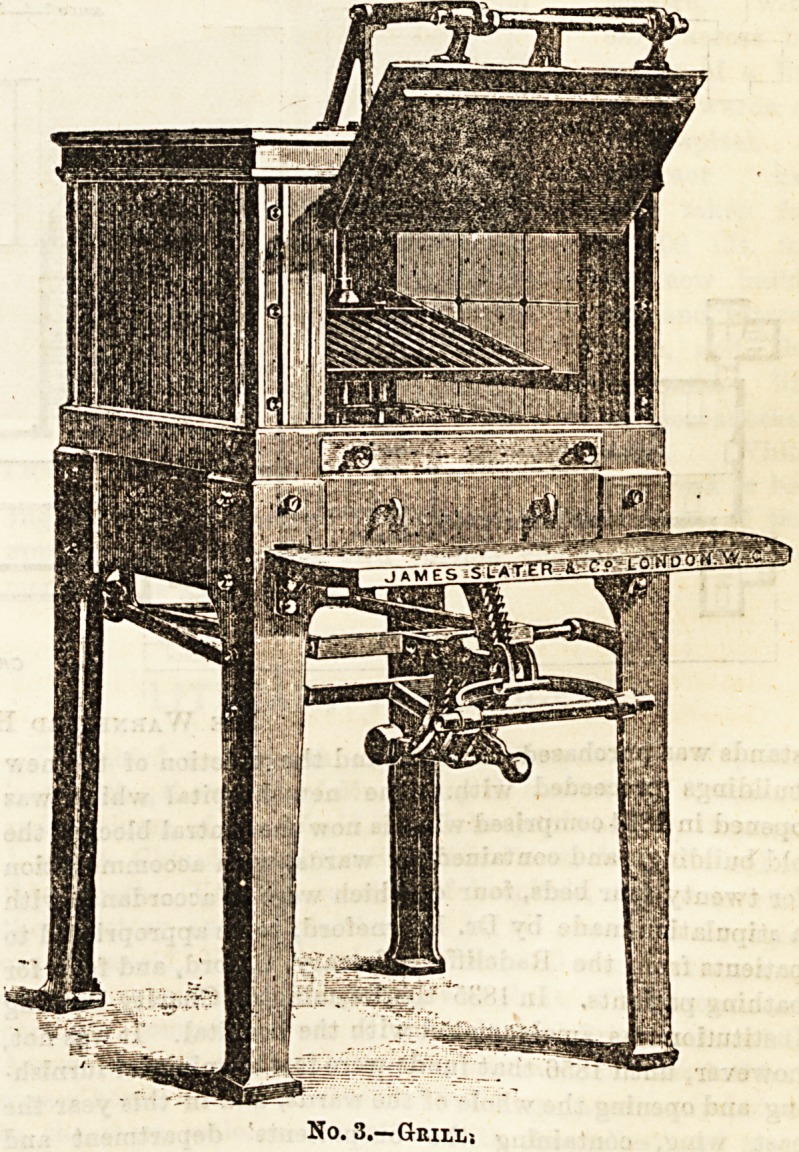# Cooking by Gas. II

**Published:** 1893-01-14

**Authors:** 


### THE INSTITUTIONAL WORKSHOP.

COOKING BY GAS.—II.

It will be remembered that in our last article we gave an illustration of a gas oven suitable for a large establishment or institution. Our second illustration shows a pastry oven,

No. 2.—PASTRY OVEN.

divided horizontally. This is also lined with glazed tiles. The heating is from an enclosed chamber below. The heat can be raised to 700 deg. The heat in the two divisions is practically identical. Such an oven is, of course, equally suitable for baking bread and cake and confectionery.

No. 3 represents a grill, which can be made to approach the flame by mechanical means, and is a most convenient contrivance. Gas exhibits superiority as fuel for grilling, more than in any other department of cookery. All smoke is avoided, and the grill can be placed in requisition at any moment without the delay frequently attendant on the preparation of a clear fire where coal is used.

We have deemed it unnecessary to give illustrations of apparatus where boiling and frying are applied. They will be familiar to most readers, and can be used in connection with a range, or as separate fittings. In most cases pipes, pierced at intervals, and bent in to circular form, are placed at certain distances apart under one covering of iron bars, upon which kettles and pans are placed. Each division of burners can be used separately.

As to the boilers, so necessary in very large establishments, these can be adjusted to an ordinary gas range by special arrangement, or they can be supplied each with independent sets of burners below. In the case where much cooking is to be performed by means of boilers, we must turn to a less direct method of applying gas in the kitchen, and one especially suitable where the kitchen is favourably placed in one of the higher storeys. We allude to the auxiliary use of steam. To realise the immense advantage of utilising steam in large establishments, a bare inspection only of a kitchen where it is in use is needed. We ourselves carefully inspected the culinary premises of the Hotel Victoria, where steam is largely used. Here the science of culinary appliance reaches its height, and the arrangements are worthy of description. The kitchen is situated on the fourth floor. The steam generator, heated by gas, is in the basement. The steam is conveyed in pipes to all parts of the kitchen. Three large boilers are ranged together, each encircled with its separate jacket of steam, and possessing separate turn-cocks, so that in one of them full boiling pitch may be maintained, while the contents of another may be simmering, and the third may not be in requisition at all. Near these boilers stands a large trough, surrounded by a steam jacket. This serves as a fish kettle, the whole of which is capable of being used, or it may be divided into several sections and then utilised to cook several kinds of fish at the same time.

On the same side of the kitchen are the vegetable ovens. These are divided like ordinary ovens into smaller or larger compartments as may be required. The doors are air-tight, and to each compartment the steam is conveyed separately, so that in cooking even one plate of peas, for instance, no waste of heat takes place. The steam enters the divisions of the oven direct, and so each becomes, for practical purposes, a steamer.

In the centre of the kitchen is placed the boiling and frying stand. Near it stands a steam jacketed trough, the water in which is maintained at a moderate temperature, so that sauces, &c., may be kept hot until required. Opposite is a large cupboard warmed by steam, for heating plates and dishes.

There is a large grill and a roasting oven. A pastry oven is placed in a subjoining department. All these last-mentioned fittings are on the principle of the illustrations we have given.

The enormous wear and tear in such a kitchen as that of the Hotel Victoria is a test of the durability of any apparatus, and we must acknowledge that Messrs. Slater have achieved a great success in having provided fittings so well

No. 3.—GRILL.

able to withstand and resist them. In our next article we propose to deal with gas-cooking appliances on a much smaller scale, including those which are suited to private rather than institutional purposes.

## Figures and Tables

**No. 2. f1:**
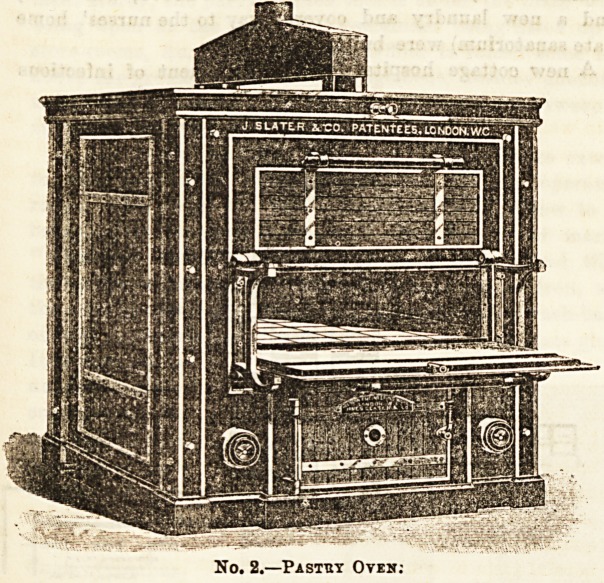


**No. 3. f2:**